# Supplementary material for: Prevalence of multimorbidity in Brazilian individuals: A population-based study
Source: PLoS One. 2024 Jan 2;19(1):e0296381. doi: 10.1371/journal.pone.0296381 (PMC10760693; doi:10.1371/journal.pone.0296381)
Supplement: Attachment — Submitted filename: PONE-D-22-31513.pdf [file pone.0296381.s001.pdf]

PLOS ONE

Prevalence of multimorbidity in Brazilian individuals: a population-based study

--Manuscript Draft--

**Manuscript Number:**	PONE-D-22-31513
**Article Type:**	Research Article
**Full Title:**	Prevalence of multimorbidity in Brazilian individuals: a population-based study
**Short Title:**	Multimorbidity in Brazilian individuals
**Corresponding Author:**	Ana Daniela Silva da Silveira, Ph.D<br>UFPA: Universidade Federal do Para<br>Belém, Pará BRAZIL
**Keywords:**	multimorbidity; noncommunicable diseases; social determinants of health; cross-sectional studies
**Abstract:**	This study aimed to estimate the prevalence of multimorbidity in Brazilian individuals and its association with sociodemographic and lifestyle factors. This cross-sectional study used data from the National Health Survey conducted in 2019 with 88,531 Brazilian adults Multimorbidity is the presence of two or more non-communicable chronic diseases. Associated factors were assessed by calculating the prevalence ratio (PR) obtained using Poisson regression with robust variance. Multimorbidity was estimated in 29.9% (95%CI: 29.33% to 30.48%) of Brazilian individuals. In the multiple models, the prevalence was high in women (PR: 1.37; 95%CI: 1.32 to 1.42), individuals over 60 years (PR: 4.26; 95%CI: 3.87 to 4.69), non-employed (PR: 1.20; 95%CI: 1.15 to 1.26), individuals with obesity (PR: 1.49; 95%CI: 1.43 to 1.56), and smokers (PR: 1.24; 95%CI: 1.19 to 1.29). This study identified a high prevalence of multimorbidity and its association with sociodemographic and lifestyle factors. The monitoring of these outcomes may support the development of policies and services.
**Order of Authors:**	Ana Daniela Silva da Silveira, Ph.D
	Jonas Eduardo Monteiro dos Santos
	Marianna de Camargo Cancela
	Dyego Leandro Bezerra de Souza
**Additional Information:**	
**Question**	**Response**
**Financial Disclosure**<br><br>Enter a financial disclosure statement that describes the sources of funding for the work included in this submission. Review the submission guidelines for detailed requirements. View published research articles from *PLOS ONE* for specific examples.<br><br>This statement is required for submission and **will appear in the published article** if the submission is accepted. Please make sure it is accurate.	The author(s) received no specific funding for this work.

**Unfunded studies**<br>Enter: *The author(s) received no specific funding for this work.*<br><br>**Funded studies**<br>Enter a statement with the following details:<br>• Initials of the authors who received each award<br>• Grant numbers awarded to each author<br>• The full name of each funder<br>• URL of each funder website<br>• Did the sponsors or funders play any role in the study design, data collection and analysis, decision to publish, or preparation of the manuscript?<br>• **NO** - Include this sentence at the end of your statement: *The funders had no role in study design, data collection and analysis, decision to publish, or preparation of the manuscript.*<br>• **YES** - Specify the role(s) played.<br><br>* typeset	
**Competing Interests**<br><br>Use the instructions below to enter a competing interest statement for this submission. On behalf of all authors, disclose any competing interests that could be perceived to bias this work—acknowledging all financial support and any other relevant financial or non-financial competing interests.<br><br>This statement is required for submission and **will appear in the published article** if the submission is accepted. Please make sure it is accurate and that any funding sources listed in your Funding Information later in the submission form are also declared in your Financial Disclosure statement.<br><br>View published research articles from *PLOS ONE* for specific examples.	The authors have declared that no competing interests exist

Powered by Editorial Manager® and ProduXion Manager® from Aries Systems Corporation

**NO authors have competing interests**<br><br>Enter: *The authors have declared that no competing interests exist.*<br><br>**Authors with competing interests**<br><br>Enter competing interest details beginning with this statement:<br><br>*I have read the journal's policy and the authors of this manuscript have the following competing interests: [insert competing interests here]*<br><br>* typeset	
**Ethics Statement**<br><br>Enter an ethics statement for this submission. This statement is required if the study involved:<br><br>• Human participants<br>• Human specimens or tissue<br>• Vertebrate animals or cephalopods<br>• Vertebrate embryos or tissues<br>• Field research<br><br>Write "N/A" if the submission does not require an ethics statement.<br><br>General guidance is provided below. Consult the submission guidelines for detailed instructions. **Make sure that all information entered here is included in the Methods section of the manuscript.**	N/A

**Format for specific study types**<br><br>**Human Subject Research (involving human participants and/or tissue)**<br>• Give the name of the institutional review board or ethics committee that approved the study<br>• Include the approval number and/or a statement indicating approval of this research<br>• Indicate the form of consent obtained (written/oral) or the reason that consent was not obtained (e.g. the data were analyzed anonymously)<br><br>**Animal Research (involving vertebrate animals, embryos or tissues)**<br>• Provide the name of the Institutional Animal Care and Use Committee (IACUC) or other relevant ethics board that reviewed the study protocol, and indicate whether they approved this research or granted a formal waiver of ethical approval<br>• Include an approval number if one was obtained<br>• If the study involved *non-human primates*, add *additional details* about animal welfare and steps taken to ameliorate suffering<br>• If anesthesia, euthanasia, or any kind of animal sacrifice is part of the study, include briefly which substances and/or methods were applied<br><br>**Field Research**<br><br>Include the following details if this study involves the collection of plant, animal, or other materials from a natural setting:<br>• Field permit number<br>• Name of the institution or relevant body that granted permission	
**Data Availability**<br><br>Authors are required to make all data underlying the findings described fully available, without restriction, and from the time of publication. PLOS allows rare exceptions to address legal and ethical concerns. See the PLOS Data Policy and FAQ for detailed information.	Yes - all data are fully available without restriction

A Data Availability Statement describing where the data can be found is required at submission. Your answers to this question constitute the Data Availability Statement and **will be published in the article**, if accepted.<br><br>**Important:** Stating 'data available on request from the author' is not sufficient. If your data are only available upon request, select 'No' for the first question and explain your exceptional situation in the text box.<br><br>Do the authors confirm that all data underlying the findings described in their manuscript are fully available without restriction?	
**Describe where the data may be found in full sentences. If you are copying our sample text, replace any instances of XXX with the appropriate details.**<br><br>• If the data are **held or will be held in a public repository**, include URLs, accession numbers or DOIs. If this information will only be available after acceptance, indicate this by ticking the box below. For example: *All XXX files are available from the XXX database (accession number(s) XXX, XXX.*).<br>• If the data are all contained **within the manuscript and/or Supporting Information files**, enter the following: *All relevant data are within the manuscript and its Supporting Information files.*<br>• If neither of these applies but you are able to provide **details of access elsewhere**, with or without limitations, please do so. For example:<br><br>*Data cannot be shared publicly because of [XXX]. Data are available from the XXX Institutional Data Access / Ethics Committee (contact via XXX) for researchers who meet the criteria for access to confidential data.*<br><br>*The data underlying the results presented in the study are available from (include the name of the third party*	All data files are available from the IBGE- Instituto Brasileiro de Geografia e Estatística database (https://www.ibge.gov.br/estatisticas/downloads-estatisticas.html)

and contact information or URL).<br>• This text is appropriate if the data are owned by a third party and authors do not have permission to share the data.<br><br>* typeset	
Additional data availability information:	Tick here if your circumstances are not covered by the questions above and you need the journal's help to make your data available.

Powered by Editorial Manager® and ProduXion Manager® from Aries Systems Corporation

Manuscript Click here to access/download;Manuscript;Manuscript.docx

Prevalence of multimorbidity in Brazilian individuals: a population-based study

Ana Daniela Silva da Silveira[1*¶], Jonas Eduardo Monteiro dos Santos[2], Marianna de Camargo Cancela[3¶], Dyego Leandro Bezerra de Souza[4¶]

[1]School of Dentistry, Federal University of Pará-UFPA, Belém, Pará, Brazil

[2]Department of Quantitative Methods in Health, Sérgio Arouca National School of Health, Rio de Janeiro, Rio de Janeiro, Brazil.

[3]Division of Population Research, National Cancer Institute, Division of Population Research, Rio de Janeiro, Brazil.

[4]Department of Public Health, Federal University of Rio Grande do Norte, Natal, Rio Grande do Norte, Brazil

*Corresponding author

Email: anadanielass@ufpa.br (ADSS)

[¶]These authors contributed equally to this study.

Abstract

This study aimed to estimate the prevalence of multimorbidity in Brazilian individuals and its association with sociodemographic and lifestyle factors. This cross-sectional study used data from the National Health Survey conducted in 2019 with 88,531 Brazilian adults Multimorbidity is the presence of two or more non-communicable chronic diseases. Associated factors were assessed by calculating the prevalence ratio (PR) obtained using Poisson regression with robust variance. Multimorbidity was estimated in 29.9% (95%CI: 29.33% to 30.48%) of Brazilian individuals. In the multiple models, the prevalence was high in women (PR: 1.37; 95%CI: 1.32 to 1.42), individuals over 60 years (PR: 4.26; 95%CI: 3.87 to 4.69), non-employed (PR: 1.20; 95%CI: 1.15 to 1.26), individuals with obesity (PR: 1.49; 95%CI: 1.43 to 1.56), and smokers (PR: 1.24; 95%CI: 1.19 to 1.29). This study identified a high prevalence of multimorbidity and its association with sociodemographic and lifestyle factors. The monitoring of these outcomes may support the development of policies and services.

Introduction

Multimorbidity is the presence of two or more chronic non-communicable diseases (NCDs) [1–5]. The literature shows that interactions among NCDs are more harmful to the quality of life than NCDs that occur separately [5, 6].

The increasing prevalence of NCDs is a worldwide phenomenon resulting from the westernization of lifestyle processes and population aging. Obesity, unhealthy eating habits, physical inactivity, and smoking are etiological factors contributing to NCDs (e.g., diabetes, high blood pressure, and cancer) [1, 6 – 9]. Data from the 2013 National Health Survey (NHS) involving 60,202 individuals showed that 23.6% of the population has

multimorbidity, which was mainly associated with sex (women), aging, low education level, smoking, and obesity [1].

The prevalence of multimorbidity in Brazilian adults directly impacts their quality of life and public health costs. In this sense, its monitoring is needed to create integral public policies focused on social and lifestyle factors [1, 6].

Therefore, this study aimed to estimate the prevalence of multimorbidity in Brazilian adults over 18 years in 2019 and its association with sociodemographic and lifestyle factors.

Methodology

This cross-sectional study analyzed data from the National Health Survey 2019 (NHS 2019) conducted by the Brazilian Ministry of Health and the Brazilian Institute of Geography and Statistics (IBGE). Data were public and had unrestricted access [10].

The NHS 2019 had a complex sampling designed in multiple stages, and sample selection was based on the IBGE master sample. The NHS 2019 assessed sociodemographic and lifestyle factors of Brazilian families in three parts: (i) the household; (ii) all household residents, focusing on sociodemographic and health information; and (iii) the selected resident, investigating information such as lifestyle, NCDs, and violence. Anthropometric measurements were also performed (sub-sample). A total of 108,525 households were visited across the country, and 94,114 interviews were performed. The non-response rate was 6.4% [10].

We analyzed 88,531 responses about the lifestyle of Brazilian individuals. Multimorbidity was defined by the presence of two or more NCDs. In this study, the condition was self-reported and based on the response to the question “Has a doctor ever

diagnosed you with (name of the condition)?". The following NCD were considered: systemic arterial hypertension, diabetes, hypercholesterolemia, heart problems (infarction, angina, or heart failure), stroke, asthma or bronchitis, arthritis, chronic back pain, work-related musculoskeletal disorders, depression, mental illness, lung diseases, cancer, and chronic kidney disease [10].

The association measures were organized according to sex, age group, ethnic group (self-reported), education level (years of schooling), occupation, physical activity, body mass index (BMI), alcohol consumption, smoking, residence area (urban or rural), Brazilian macro-regions (north, northeast, southeast, south, and midwest), private health insurance, and medical care in the last year [10].

Alcohol consumption was classified as abstaining, moderate (two drinks a day for men and one drink a day for women), or excessive (five drinks or more in a day for men and four drinks or more in a day for women). Smoking was classified as never smoked, ex-smoker, or smoker [11]. For BMI, we used the World Health Organization (WHO) classification: underweight (BMI < 18.5 kg/m^2), normal weight (BMI between 18.5 and 24.5 kg/m^2), overweight (BMI between 25 and 29.9 kg/m^2), and obesity (BMI $\geq$ 30 kg/m^2). Physical activity was classified as none, not enough, or enough, according to the total number of minutes per week dedicated to physical exercise, sports, leisure, efforts at work, commuting from work, or other displacements and household chores that involved physical effort. The WHO classifies the level of physical activity as moderate (75 to 150 minutes) or intense (150 to 300 minutes) [12].

Poisson regression with robust variance verified associations between multimorbidity and independent variables; data regarding the prevalence ratio (PR) of multimorbidity and 95% confidence intervals were also estimated. The Wald test assessed the significance of the models with p-value <0.05. All analyzes were performed using the

statistical package STATA (Stata Corp. Inc. TX, USA, version 14) and adjusted for complex sample design.

The National Research Ethics Commission approved the NHS 2019 in August 2019 (registry no. 3,529,376).

Results

The NHS 2019 involved 88,531 individuals corresponding to an estimated 159,171,311 individuals. A total of 53.16% individuals were women; 21.61% had advanced age (> 60 years); and 12.59% were smokers. The prevalence of multimorbidity was 29.9% with an estimated frequency of 47,598,091 Brazilian individuals (Table 1).

Table 1. Estimated prevalence of multimorbidity according to sociodemographic characteristics, social determinants, lifestyle factors, and use of health services.

Variables	N	% (95%CI[a])	pb
**Sex**			
Men	16,894,158	22.66 (21.94 to 23.39)	<0.001
Women	30,703,933	36.29 (35.48 to 37.10)	
**Age group**			
18 to 29	3,271,881	9.30 (8.51 to 10.15)	<0.001
30 to 39	5,425,932	16.24 (15.30 to 17.22)	
40 to 49	8,048,579	27.82 (26.54 to 29.14)	
50 to 59	11,344,470	41.63 (40.07 to 43.21)	
60+	19,507,228	56.71 (55.61 to 57.80)	
**Ethnic group**			
White	22,493,190	32.67 (31.79 to 33.56)	<0.001
Non-white	25,104,901	27.80 (27.09 to 25.51)	
**Education level**			
0 to 3	1,348,680	43.79 (40.60 to 47.02)	<0.001
4 to 7	21,082,639	37.81 (36.84 to 38.79)	
8 to 10	12,641,951	22.12 (21.30 to 22.96)	
11 or more	9,250,973	25.68 (24.54 to 26.85)	
**Employed**			
Yes	21,986,724	22.55 (21.86 to 23.24)	<0.001
No	25,611,367	41.54 (40.63 to 42.47)	
**Physical activity**			
None	13,078,640	38.10 (36.90 to 39.31)	<0.001
Not enough	8,307,504	32.85 (31.45 to 34.28)	
Enough	26,211,947	26.33 (25.66 to 27.02)	
**BMI**			
Normal weight	14,418,484	23.11 (22.33to 23.91)	<0.001
Underweight	833,051	24.43 (20.86 to 28.38)	
Overweight	18,438,237	31.64 (30.72 to 32.58)	
Obesity	13,674,174	40.85 (39.56 to 42.15)	
**Alcohol consumption**			
Abstaining	31,376,263	34.09 (33.33 to 34.85)	<0.001
Moderate	10,800,988	27.03 (26.02 to 28.06)	
Excessive	5,420,840	19.96 (18.90 to 21.06)	
**Smoking**			
Never smoked	24,748,533	25.57 (24.89 to 26.27)	<0.001
Ex-smoker	17,141,204	40.46 (39.30 to 41.63)	
Smoker	5,708,354	28.49 (27.04 to 30.00)	
**Residence area**			
Urban	41,789,954	30.47 (29.82 to 31.11)	<0.001
Rural	5,808,137	26.40 (25.30 to 27.54)	
**Brazilian region**			
North	2,695,262	21.57 (20.52 to 22.66)	<0.001
Northeast	11,260,993	26.74 (25.88 to 27.63)	
Southeast	22,512,241	32.56 (31.47 to 33.66)	
South	7,826,332	33.48 (32.19 to 34.81)	
Midwest	3,303,263	27.42 (26.12 to 28.76)	

**Private health insurance**			
Yes	15,579,447	33.02 (31.94 to 34.12)	<0.001
No	32,018,644	28.59 (27.97 to 29.22)	
**Medicines used in the last year**			
Yes	44,652,577	34.78 (34.11 to 35.45)	<0.001
No	2,945,515	9.57 (8.87 to 10.31)	

[a]CI: confidence interval; [b]*p*: Wald test

The most frequent NCDs in both sexes were systemic arterial hypertension, chronic back pain, and hypercholesterolemia. A high prevalence of multimorbidity was observed among women (36.29%; 95%CI: 35.48 to 37.10) and individuals over 60 years (56.71%; 95%CI: 55.61 to 57.80). The results showed that age exerted a dose-response effect on the occurrence of multimorbidity (Fig 1).

Fig 1. Distribution of NCDs in individuals with multimorbidity according to sex.

Regarding sociodemographic factors, a high prevalence of multimorbidity was found in individuals with low education level (over three years of schooling) (43.79%; 95%CI: 40.60 to 47.02) and non-employed (41.54%; 95%CI: 40.63 to 42.47).

Regarding lifestyle factors, smokers (40.46%; 95%CI: 39.30 to 41.63) and individuals with obesity (40.85%; 95%CI: 39.56 to 42.15) had the highest estimated values for multimorbidity. The prevalence of multimorbidity according to independent variables is described in Table 2. All variables were included in the multiple regression model.

Table 2. Prevalence of multimorbidity in Brazilian individuals according to lifestyle and social factors.

Variables	PR[a] non-adjusted	95%CI[b]	p[c]	PR adjusted	95%CI	p
**Sex**						
Men	1.00			1.00		
Women	1.60	1.54 to 1.66	< 0.001	1.37	1.32 to 1.42	< 0.001
**Age group**						
18 to 29	1.00			1.00		
30 to 39	1.74	1.57 to 1.94	< 0.001	1.64	1.47 to 1.82	< 0.001
40 to 49	2.99	2.71 to 3.30		2.62	2.37 to 2.90	
50 to 59	4.47	4.07 to 4.92		3.60	3.26 to 3.97	
60+	6.09	5.58 to 6.66		4.26	3.87 to 4.69	
**Ethnic group**						
White	1.00			1.00		
Non-white	1.74	1.13 to 1.22	< 0.001	1.00	0.97 to 1.04	0.770
**Education level**						
0 to 3	1.00			1.00		
4 to 7	0.86	0.80 to 0.93	< 0.001	0.99	0.92 to 1.06	< 0.001
8 to 10	0.51	0.46 to 0.55		0.90	0.83 to 0.97	
11 or more	0.58	0.54 to 0.64		0.94	0.86 to 1.02	
**Employed**						
Yes	1.00			1.00		
No	1.84	1.77 to 1.91	< 0.001	1.20	1.15 to 1.26	< 0.001
**Physical activity**						
None	1.00			1.00		
Not enough	1.25	1.19 to 1.31	< 0.001	1.03	0.98 to 1.08	0.350
Enough	1.45	1.39 to 1.50		1.02	0.98 to 1.06	
**BMI[d]**						
Normal weigh	1.00			1.00		
Underweight	1.06	0.90 to 1.23	< 0.001	1.06	0.90 to 1.24	< 0.001
Overweight	1.37	1.31 to 1.43		1.24	1.19 to 1.29	
Obesity	1.77	1.69 to 1.85		1.49	1.43 to 1.56	
**Alcohol consumption**						
Abstaining	1.00			1.00		
Moderate	0.79	0.76 to 0.83	< 0.001	0.91	0.87 to 0.96	< 0.001
Excessive	0.58	0.55 to 0.62		0.98	0.93 to 1.04	
**Smoking**						
Never smoked	1.00			1.00		
Ex-smoker	1.58	1.52 to 1.64	< 0.001	1.24	1.19 to 1.29	< 0.001
Smoker	1.11	1.05 to 1.18		1.16	1.09 to 1.22	
**Residence area**						
Urban	1.00			1.00		
Rural	1.53	1.10 to 1.21	< 0.001	1.13	0.99 to 1.08	0.134
**Brazilian region**						
North	1.00			1.00		
Northeast	1.24	1.17 to 1.31	< 0.001	1.10	1.04 to 1.16	< 0.001
Southeast	1.51	1.42 to 1.60		1.21	1.14 to 1.28	

South	1.55	1.46 to 1.65		1.23	1.16 to 1.32	
Midwest	1.27	1.18 to 1.36		1.14	1.07 to 1.22	
**Private health insurance**						
Yes	1.00			1.00		
No	1.15	1.11 to 1.20	< 0.001	1.08	1.03 to 1.12	< 0.001
**Medicines used in the last year**						
Yes	1.00			1.00		
No	3.63	3.36 to 3.93	< 0.001	2.59	2.39 to 2.80	< 0.001

[a]PR: Prevalence ratio; [b]CI: confidence interval; [c]*p*: Wald test; [d]BMI: body mass index.

The multiple regression showed that the prevalence of multimorbidity among women (PR: 1.37; 95%CI: 1.32 to 1.42) was 37% higher than in men, regardless of other variables. In the adjusted analysis, the prevalence significantly increased with age: multimorbidity was 3.26-fold higher in individuals over 60 years (PR: 4.26; 95%CI: 3.87 to 4.69) than in individuals aged between 18 and 29 years.

Individuals with obesity (PR: 1.49; 95%CI: 1.43 to 1.56) and who smoked (PR: 1.16; 95%CI: 1.09 to 1.23) also had a higher prevalence of multimorbidity than individuals with normal weight and those who never smoked, respectively.

The southeast region (PR: 1.23; 95%CI: 1.16 to 1.32) presented the highest prevalence of multimorbidity compared with the other Brazilian regions.

Discussion

The prevalence of multimorbidity in Brazilian individuals was 29.9%, which was higher than in previous Brazilian surveys (23.6%) [1–4]. Moreover, a high prevalence was found in women and individuals over 60 years, corroborating with worldwide trends for NCDs [5–13].

Systemic arterial hypertension, chronic back pain, and hypercholesterolemia were the most prevalent NCDs in both sexes, as observed in NHS 2013 [1]. This result emphasizes the need for attention, especially within the scope of public health.

Regarding associations between sociodemographic factors and multimorbidity, a study conducted with 5,074,227 individuals in the Netherlands observed that unemployed individuals were more likely to have at least three than those employed [1]. These findings corroborated our results since non-employed individuals had a higher prevalence of multimorbidity, regardless of age, sex, or education level.

Individuals with private health insurance had a higher prevalence of multimorbidity than those with no private health insurance. Moreover, individuals who had at least one medical appointment in the year before the survey showed a 2.59-fold increase in the prevalence of multimorbidity compared with those who did not seek a doctor. Access to health services and the accurate diagnosis of NCDs may be easier in private than in public health, favoring prevention. These results may be justified by the question asked during the survey ("Has a doctor ever diagnosed you with (name of the condition)?"), which probably influenced the number of cases of multimorbidity among individuals who had easy access to medical care.

The north region of Brazil, which is the least economically developed, had the lowest prevalence of multimorbidity compared with other regions. In contrast, the southeast and south regions (most economically developed regions) had the highest prevalence of multimorbidity. In addition, the north region of Brazil has specific geographic characteristics (i.e., many rivers, dense vegetation, and a wide territory with low population density) that may justify the difficult access to health services [1, 2]. A survey conducted by the Institute of Studies for Health Policies [1] showed that many older individuals from the north region never performed a blood test to measure

cholesterol or blood glucose compared with other regions of Brazil. The literature and our results suggested low access to diagnosis and screening of NCDs in the northern region of Brazil.

Lifestyle factors were significantly associated with multimorbidity. We found a high prevalence of multimorbidity in individuals with obesity (49%) and smokers (24%) compared with those with normal weight and who never smoked, respectively. Similarly, a 20-year cohort study conducted in the United Kingdom with 4,510 individuals aged between 15 and 55 years found an increasing prevalence of multimorbidity. Smoking and obesity were strong independent predictors of multimorbidity [3].

The association between multimorbidity and lifestyle should be a strategy for creating health policies to prevent and treat NCDs in adults and older individuals. These variables may also be important for a comprehensive and multidisciplinary planning, including holistic health and social care, and would reduce the burden of multimorbidity and disadvantages related to lack of prevention, treatment, or follow-up of individuals.

The Brazilian public health system has public policies to treat and control NCDs, such as the Network of Non-Communicable Chronic Diseases, the National Program for Attention to Arterial Hypertension and Diabetes Mellitus, and many other programs created especially for women and older individuals. Based on this context and the correlation with other diseases, it is possible to treat or control NCDs using lifestyle and therapeutic interventions, justifying the public investment in these policies [1–4].

The present study had some limitations. The self-reported diagnosis of NCDs may have underestimated the prevalence of multimorbidity. The cross-sectional design may have also precluded the analysis of causal relationships. Nevertheless, the use of a population database and complex statistical analyses allowed us to extrapolate the results for the entire Brazilian population.

Conclusion

The prevalence of multimorbidity in Brazilian individuals has been increasing since the NHS 2013. Systemic arterial hypertension, chronic back problems, and hypercholesteremia were the most prevalent NCDs in both sexes.

The prevalence of multimorbidity was associated with sociodemographic and lifestyle factors. Therefore, these characteristics are essential to health services for planning strategies to prevent, treat, and control multimorbidity.

Acknowledgments

References

1. de Carvalho JN de Camargo Cancela M Leandro D de Souza B. Lifestyle factors and high body mass index are associated with different multimorbidity clusters in the Brazilian population. PLoS ONE. 2018;13.
2. de Carvalho JN Roncalli ÂG de Camargo Cancela M de Souza DLB. Prevalence of multimorbidity in the Brazilian adult population according to socioeconomic and demographic characteristics. PLoS ONE. 2017;12.
3. Nunes BP Chiavegatto Filho ADP Pati S Cruz Teixeira DS Flores TR Camargo-Figuera FA et al. Contextual and individual inequalities of multimorbidity in Brazilian adults: A cross-sectional national-based study. BMJ Open. 2017;7.
4. Rzewuska M de Azevedo-Marques JM Coxon D Zanetti ML Zanetti ACG Franco LJ et al. Epidemiology of multimorbidity within the Brazilian adult general population: Evidence from the 2013 National Health Survey (PNS 2013). PLoS ONE. 2017;12:1–13.
5. World Health Organization Alwan A Armstrong T Bettcher D Branca F Chisholm D et al. Global status report on noncommunicable diseases 2010. World Health Organization; 2011.
6. Afshar S Roderick PJ Kowal P Dimitrov BD Hill AG. Multimorbidity and the inequalities of global ageing: A cross-sectional study of 28 countries using the World Health Surveys. BMC Public Health. 2015;15.
7. Katikireddi SV Skivington K Leyland AH Hunt K Mercer SW. The contribution of risk factors to socioeconomic inequalities in multimorbidity across the lifecourse: A longitudinal analysis of the twenty-07 cohort. BMC Medicine. 2017;15.
8. Feter N Leite JS Umpierre D Caputo EL Rombaldi AJ. Multimorbidity and leisure-time physical activity over the life course: a population-based birth cohort study. BMC Public Health. 2021;21.

9. Asogwa OA Boateng D Marzà-Florensa A Peters S Levitt N van Olmen J et al. Multimorbidity of non-communicable diseases in low-income and middle-income countries: A systematic review and meta-analysis. BMJ Open. 2022;12.
10. IBGE. Pesquisa Nacional de Saúde 2019 - Informações sobre domicílios acesso e utilização dos serviços de saúde : Brasil grandes regiões e unidades da federação. Rio de Janeiro: IBGE; 2020.
11. World Cancer Research Fund American Institute for Cancer Research. Body fatness and weight gain and the risk of cancer. 2018.
12. World Health Organization. WHO GUIDELINES ON PHYSICAL ACTIVITY AND SEDENTARY BEHAVIOUR. Geneva Switzerland: World Health Organization; 2020.
13. Nunes BP Batista SRR de Andrade FB de Souza Junior PRB Lima-Costa MF Facchini LA. Multimorbidity: The Brazilian Longitudinal Study of Aging (ELSI-Brazil). Revista de Saude Publica. 2018;52.
14. Delpino FM Wendt A Crespo PA Blumenberg C Teixeira DS da C Batista SR et al. Occurrence and inequalities by education in multimorbidity in Brazilian adults between 2013 and 2019: evidence from the National Health Survey. Revista Brasileira de Epidemiologia. 2021;24.
15. Shi X Lima SM da S Mota CM de M Lu Y Stafford RS Pereira CV. Prevalence of Multimorbidity of Chronic Noncommunicable Diseases in Brazil: Population-Based Study. JMIR Public Health and Surveillance. 2021;7:e29693.
16. Cimarras-Otal C Calderón-Larrañaga A Poblador-Plou B González-Rubio F Gimeno-Feliu LA Arjol-Serrano JL et al. Association between physical activity multimorbidity self-rated health and functional limitation in the Spanish population. BMC Public Health. 2014;14.
17. Shi J Guo Y Li Z Liang Z Pan L Yu Y et al. Sociodemographic and behavioral influences on multimorbidity among adult residents of northeastern China. BMC Public Health. 2022;22.
18. Romano E Ma R Vancampfort D Firth J Felez-Nobrega M Haro JM et al. Multimorbidity and obesity in older adults from six low- and middle-income countries. Preventive Medicine. 2021;153:106816.
19. Huaquiá-Diáz AM Chalán-Dávila TS Carrillo-Larco RM Bernabe-Ortiz A. Multimorbidity in Latin America and the Caribbean: A systematic review and meta-analysis. BMJ Open. 2021;11.
20. Yildiz B Schuring M Knoef MG Burdorf A. Chronic diseases and multimorbidity among unemployed and employed persons in the Netherlands: A register-based cross-sectional study. BMJ Open. 2020;10.
21. Bezerra de Souza DL Oliveras-Fabregas A Espelt A Bosque-Prous M de Camargo Cancela M Teixidó-Compañó E et al. Multimorbidity and its associated factors among adults aged 50 and over: A cross-sectional study in 17 European countries. PLoS ONE. 2021;16 2 Febuary.
22. Puth MT Weckbecker K Schmid M Münster E. Prevalence of multimorbidity in Germany: Impact of age and educational level in a cross-sectional study on 19 294 adults. BMC Public Health. 2017;17.
23. Yao SS Cao GY Han L Chen ZS Huang ZT Gong P et al. Prevalence and patterns of multimorbidity in a nationally representative sample of older chinese: Results from the china health and retirement longitudinal study. Journals of Gerontology - Series A Biological Sciences and Medical Sciences. 2020;75:1974–80.

24. Rocha R Camargo M Falcão L Silveira M Thomazinho G. A Saúde na Amazônia Legal Evolução Recente e Desafios em Perspectiva Comparada. 2021.
25. Garnelo L Lima JG Rocha ESC Herkrath FJ. Acesso e cobertura da Atenção Primária à Saúde para populações rurais e urbanas na região norte do Brasil. Saúde em Debate. 2018;42:81–99.
26. Brasil Departamento de Ações Programáticas Estratégicas. Política nacional de atenção integral à saúde da mulher : princípios e diretrizes. Editora MS; 2004.
27. Brasil Ministério da Saúde Secretaria de Assistência à Saúde. Diretrizes para o cuidado das pessoas com doenças crônicas nas redes de atenção à saúde e nas linhas de cuidado prioritárias. 2013.
28. Brasil Ministério da Saúde Secretaria de Assistência à Saúde. Redes estaduais de atenção à saúde do idoso : guia operacional e portarias relacionadas. Editora MS; 2002.
29. Coelho LP da Motta LB Caldas CP. Elderly care network: Facilitating factors and barriers to implementation. Physis. 2018;28.

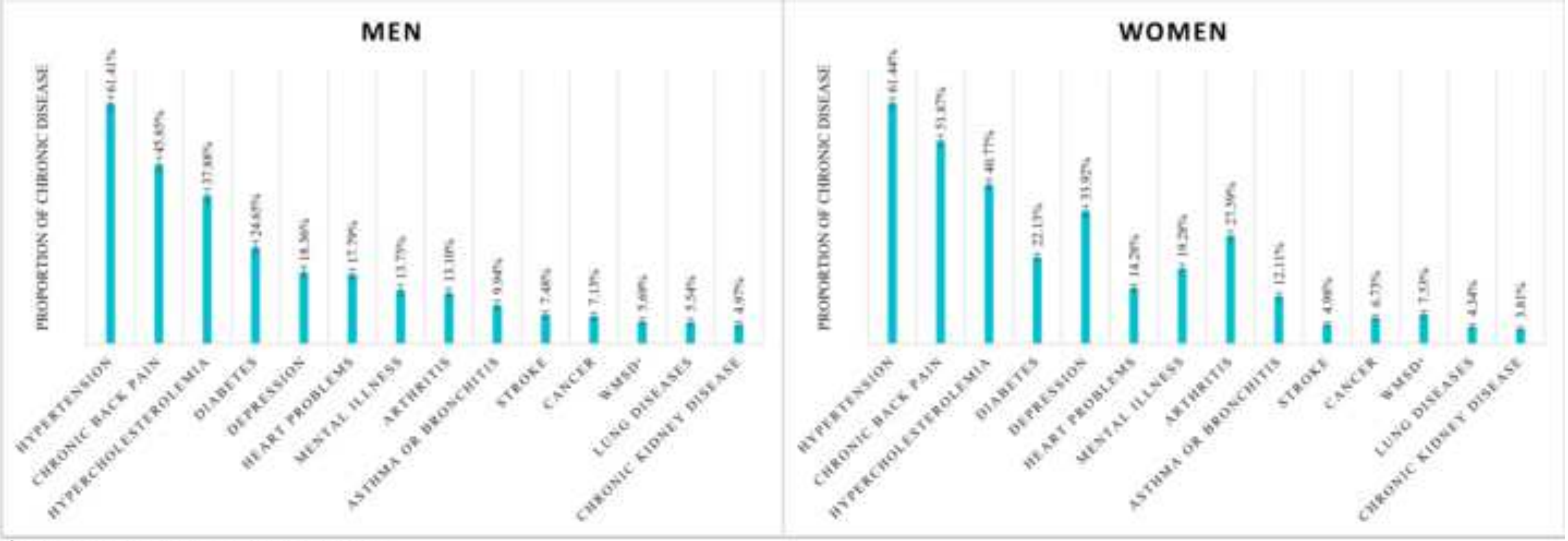

*WMSD: Work-related musculoskeletal disorders
