# Supplementary material for: Prevalence of multimorbidity in Brazilian individuals: A population-based study
Source: PLoS One. 2024 Jan 2;19(1):e0296381. doi: 10.1371/journal.pone.0296381 (PMC10760693; doi:10.1371/journal.pone.0296381)
Supplement: Attachment — Submitted filename: Response to Reviewers.docx [file pone.0296381.s002.docx]

Belém/Pará/Brazil, April 18, 2023

Dear Editor,

The authors carefully revised the manuscript "Prevalence of multimorbidity in Brazilian individuals: a population-based study" for consideration by the Editorial Committee of the journal PLOS ONE.

According to the corrections suggested by the reviewers, we present the changes in the manuscript:

Reviewer #1:

In the Methodology Section please provide the following :

• Basic description of multistage sampling used and clarification at what level(s) was the National Health Survey (NHS) representative;

Suggestion accepted.

• Detailed description of all ethical considerations of the NHS;

Suggestion accepted.

• Explanation on how NHS data became public with unrestricted access?

Suggestion accepted.

• Explanation why did you analyze 88,531 responses and why multimorbidity was defined by the presence of two or more NCDs and later in the text you use the term condition?

Suggestion accepted

• Description of all independent variables used (for example: level of education, what do working status and minutes of physical activity mean);

Suggestion accepted.

• Description of prerequisites for and procedure of the Poisson regression used.
Suggestion accepted.

For the Results Section please do the following:

• As Prevalence Ratio (PR) and Odds Ratio (ORs) are not the same thing and logistic regression coefficient B, Exp(B) and Wald test have values with regard to p value (Sig.), perform analyses again and use clearly defined statistical marks in the presenting tables.

In this study, we chose to carry out the Poisson regression model, the estimated measure used was the IRR (Incidence Rate Ratio) and the Wald test was used as post-estimation of the Poisson model. We do not use logistic regression.

Reviewer #2: The manuscript addresses an emerging theme in the literature. However, the rationale is very superficial. The methods need more detail and the adjusted analysis seems to me to have many flaws, there is a need to review the adjustments. There is no focus on evaluating MM associated with any specific factor and this makes the manuscript much more superficial in other points, such as the discussion that needs greater depth. Comments were made in the attached text.

INTRODUCTION

1- The rationale of the study is not enough. What is the data innovation for the area and literature?

Suggestion accepted

2- Check the references –

The references have been adjusted

METODOLOGY

1- Review, as the list of PNS diseases is longer. (line 66)

We've added a figure to make it easier to understand.

2- Explain all the variables and their operationalization (line 67)
   Its done
3- Mention which statistical procedures for the description of data (line 83)

Suggestion accepted

RESULTS

1- Table 1 - Is the p-value for assessing differences between variable categories? It would leave only 95%CI without presenting a statistical test. According to this review
2- Table 2 (legenda) - The adjustments were not explained in the methods. Got corrected

DISCUSSION

1- LINE 134 - Need to show association results

Suggestion accepted

2- Line 181 - Limitations were pointed out, but poorly discussed/defended. It is necessary to show what was the conduct to minimize possible biases.

Suggestion accepted

CONCLUSION

1- It doesn't seem correct. In the present study, no comparison was made between surveys.

Suggestion accepted

Sincerely, the authors.
