# Supplementary material for: Prevalence of multimorbidity in Brazilian individuals: A population-based study
Source: PLoS One. 2024 Jan 2;19(1):e0296381. doi: 10.1371/journal.pone.0296381 (PMC10760693; doi:10.1371/journal.pone.0296381)
Supplement: Attachment — Submitted filename: Review for PONE-D-22-31513R1.docx [file pone.0296381.s003.docx]

Dear Editor,

Thank you for inviting me again to review the corrected manuscript submitted to your distinguished journal (PONE-D-22-31513R1: Prevalence of multimorbidity in Brazilian individuals: a population-based study)

I appreciate efforts of the authors to address global health issue such as multimorbidity and present results of the national survey, but in the manuscript there are several missing or poorly described facts for which I would return the manuscript for another revision.

Please find below my remarks:

1. On the Page 4, Line 78 – Authors write: “We analyzed 88,531 responses about the lifestyle of Brazilian individuals corresponding to the responses of people over 18 years old.”; and,
2. on the Page 6, Line 117 authors write: “The NHS 2019 involved 88,531 individuals corresponding to an estimated 159,171,311 individuals”; and,
3. present on the Page 7, Table 1. Estimated prevalence of multimorbidity according to sociodemographic characteristics, social determinants, lifestyle factors, and use of health services, with the number of participants of 47,598,091; and,
4. write statement, as an answer to the reviewer, that “In this study, we chose to carry out the Poisson regression model, the estimated measure used was the IRR (Incidence Rate Ratio) and the Wald test was used as post-estimation of the Poisson model. We do not use logistic regression”.

To me it is not clear:

1. what is the number of participants covered by the authors analyses?
2. why and how did authors use Incidence Rate Ratio (IRR) for data from typical prevalence study such as National Health Survey?

(an **incidence rate ratio** allows us to compare the incident rate between two different groups, exposed vs.non-exposed)

3. where did they present assumptions for the use of Poisson regression?

(two out of four assumptions should be that **the distribution of counts follows a Poisson distribution and mean and variance of the model are equal)**

4. **why they interpret obtained ratios as high not giving belonging comparison (for example if IRR Greater than 1:** This indicates that the incident rate is greater in an exposed group compared to an unexposed group).

With kind regards.
