# Supplementary material for: Prevalence of multimorbidity in Brazilian individuals: A population-based study
Source: PLoS One. 2024 Jan 2;19(1):e0296381. doi: 10.1371/journal.pone.0296381 (PMC10760693; doi:10.1371/journal.pone.0296381)
Supplement: Attachment — Submitted filename: Response to Reviewers.docx [file pone.0296381.s004.docx]

11 de outubro de 2023

Dear Editor,

We appreciate the new suggestions for publication. The authors carefully reviewed the manuscript "Prevalence of multimorbidity in Brazilian individuals: a population-based study". Our responses to the considerations of **Reviewer n#1 and Reviewer n#2** are listed below

**Dear Reviewer n#1**, we accepted your suggestions and made the necessary adjustments according to your guidelines. We are grateful for that and have provided the answers to your questions below:

The Reviewer #1 says:

"Please find below my remarks:

1.	On the Page 4, Line 78 – Authors write: “We analyzed 88,531 responses about the lifestyle of Brazilian individuals corresponding to the responses of people over 18 years old.”; and,

2.	on the Page 6, Line 117 authors write: “The NHS 2019 involved 88,531 individuals corresponding to an estimated 159,171,311 individuals”; and,

3.	present on the Page 7, Table 1. Estimated prevalence of multimorbidity according to sociodemographic characteristics, social determinants, lifestyle factors, and use of health services, with the number of participants of 47,598,091; and,

4.	write statement, as an answer to the reviewer, that “In this study, we chose to carry out the Poisson regression model, the estimated measure used was the IRR (Incidence Rate Ratio) and the Wald test was used as post-estimation of the Poisson model. We do not use logistic regression”."

A: All suggestions accepted and text corrected as explained below

The Reviewer #1 ask:

1.	what is the number of participants covered by the authors analyses?

A: The number of responses analyzed in this research was 88,531 (On page 4, line 78 and page 6, line 117), corresponding to responses from individuals aged over 18 years. The SVY module for complex statistics made it possible to estimate these values for the total number of responses (159,171,311; page 6, line 117) and, of this total, the number of people in the category "with multiborbity" (people with 2 or more chronic diseases) is 47,598,091, represented in Table 1 (Page 7, Table 1).

Reviewer #1 - 2&3:

•	write statement, as an answer to the reviewer, that “In this study, we chose to carry out the Poisson regression model, the estimated measure used was the IRR (Incidence Rate Ratio) and the Wald test was used as post-estimation of the Poisson model. We do not use logistic regression”. and

2.	why and how did authors use Incidence Rate Ratio (IRR) for data from typical prevalence study such as National Health Survey? (an incidence rate ratio allows us to compare the incident rate between two different groups, exposed vs. non-exposed). and

3. where did they present assumptions for the use of Poisson regression? (two out of four assumptions should be that the distribution of counts follows a Poisson distribution and mean and variance of the model are equal)

A: The classic Poisson probability distribution is applied to count data to model rates over time and predict whether the mean and variance are equal. However, in situations of underdispersion or overdispersion of the data – in which the mean and variance are not equal – it is possible to make adaptations to the classical model, using link functions. In this case, using Poisson modeling fits the binary result data using the logarithmic function to linearize the relationship between the result and the independent variables. It's the same assumption as logistic regression, but uses the logit link function. In the classic use of the Poisson distribution, the measure of association, after beta exponentiation, is the IRR, like the incidence rate of a given event. However, in the linearization process with the logarithmic function, the classic association measure can be interpreted as a prevalence ratio, as it is cross-sectional data.

Reviewer #1 - 4:

4.	why they interpret obtained ratios as high not giving belonging comparison (for example if IRR Greater than 1: This indicates that the incident rate is greater in an exposed group compared to an unexposed group).

A: Suggestion accepted and text corrected (Page 8, Line 127 and 135)

**Dear Reviewer n#2,** we accepted your suggestions and made the necessary adjustments according to your guidelines. We are grateful for that.
